# Demographic and environmental factors associated with canine cutaneous squamous cell carcinoma development in Australia: a retrospective matched case-control analysis

**DOI:** 10.1007/s11259-026-11595-5

**Published:** 2026-10-24

**Authors:** Jingwei Jennifer Zhang, Haileyesus Dejene, José Luis Granados-Soler, Joerg Henning, Rachel Allavena

**Affiliations:** School of Veterinary Science, The University of Queensland, Gatton Qld 4343, Gatton Qld, 4343 Australia

**Keywords:** Australia, Canine, Cutaneous squamous cell carcinoma, Spatial epidemiology, Ultraviolet radiation, VetCompass

## Abstract

Cutaneous squamous cell carcinoma (cSCC) is a common canine skin tumour that occurs more frequently in high ultraviolet (UV) environments. Despite Australia’s consistently high UV exposure, large-scale epidemiological studies on canine cSCC are limited. This study investigates the frequency of canine cSCC in Australia, its geographic patterns relative to UV index, and its association with animal-level risk factors. A matched case-control study was conducted using records from the VetCompass database from 1st January 2014 to 31st December 2023. In total, 295 histopathologically confirmed cSCC cases and 1,180 matched controls were included. Frequency statistics of cSCC and animal-level risk factors associated with cSCC were analysed and spatial patterns were mapped. The highest proportions of cases were recorded in Queensland (39.7%) and New South Wales (39.3%), with increased frequency along coastal areas of both states. cSCC cases occurred most frequently on body regions caudal to the thorax (54.9%), in senior dogs (58.6%), in desexed males (45.1%), and in dogs with white mixed coat colour (43.7%). In the final multivariable model, senior (seven to 11 years old) and geriatric (12 + years old) dogs had significantly higher odds of cSCC than mature (two to six years old) dogs (odds ratio = 7.4 and 4.8, respectively; *p* ≤ 0.001). Risk was also markedly elevated in Dalmatians, bulldogs, Staffordshire terriers, and schnauzers compared with mixed breeds. Dogs with white or white mixed coats had higher odds of cSCC than dark-coated dogs (*p* ≤ 0.001). cSCC demonstrated anatomical and geographic patterns consistent with chronic UV exposure, with age, breed, and coat pigmentation emerging as risk factors.

**Supplementary Information:**

The online version contains supplementary material available at https://doi.org/10.1007/s11259-026-11595-5.

## Introduction

Neoplasms account for 15 to 30% of deaths in dogs, making them a major cause of disease-related mortality (Brønden et al. 2010). Their incidence has increased in recent years, with cutaneous tumours reported as the most common type of neoplasia in dogs in many countries (Dobson et al. 2002; Gamlem et al. 2008; Grüntzig et al. 2015; Śmiech et al. 2023). Cutaneous squamous cell carcinoma (cSCC) is a malignant tumour arising from keratinocytes in the skin (dos Santos et al. 2023; Łojszczyk et al. 2021; Seok and Lee 2024). It represents approximately 5% of canine skin tumours worldwide, has higher prevalence in tropical countries, and has high local invasiveness but low metastatic potential (Alves et al. 2022; Bastianello 1983; Chikweto et al. 2011; Kok et al. 2019; Kukolj et al. 2021; Mukaratirwa et al. 2005; Santos et al. 2020). Despite having low mortality rates, its prevalence and clinical impact make cSCC an important dermatological and oncological concern in veterinary practice.

UV light is a well-established carcinogenic factor in canine cSCC pathogenesis. Multiple studies worldwide have described associations between chronic solar radiation exposure and squamous cell carcinoma (SCC) development in dogs (Hargis et al. 1977; Madewell et al. 1981; Nikula et al. 1992; Subapriya et al. 2025). Geographically, higher incidences of canine SCC are often found in regions with high average UV intensity (García-Reynoso et al. 2025). Australia receives a very high amount of UV radiation due to being located close to the equator (Gies et al. 2004). UV radiation can be quantitatively measured through the UV index developed by the Australian Radiation Protection and Nuclear Safety Agency (ARPANSA). At noon, UV index measurements above 11 are considered extreme (Wright and Norval 2021). The average UV index measured in Australia during peak hours in summer is often greater than 12 to 14, potentially reaching 16 to 17 in northern regions (Gies et al. 2004). However, previous research comparing canine SCC prevalence between tropical Queensland and Melbourne in 1983 failed to identify clear links between regional UV differences and cSCC prevalence (Ladds et al. 1983). This apparent inconsistency highlights the need for an updated analytical overlook.

Animal-specific demographic factors such as age, breed and colour impact cSCC occurrence. Although patients of all ages can potentially develop cSCC, the most severely affected population are mature and geriatric dogs (Er and Sutton 1989; Martins et al. 2022). The average age for canine patients to be diagnosed with squamous cell carcinoma falls between seven and nine years, with a small degree of variation amongst different studies (Bastianello 1983; dos Santos et al. 2023; Handrashekaraiah et al. 2011; Rothwell et al. 1987; Strafuss et al. 1976; Villamil et al. 2011). On the other hand, a long list of predisposed breeds has been identified with inconsistencies across studies. Typical at-risk breeds include Dalmatians, Staffordshire bull terriers, schnauzers, Jack Russell terriers, bulldogs and Rottweilers, with many other breeds also occasionally mentioned (dos Santos et al. 2023; Er and Sutton 1989; Fonti et al. 2025; Grüntzig et al. 2016; Kok et al. 2019; Rothwell et al. 1987; Tompkins et al. 2020; Villamil et al. 2011). Contrastingly, boxers are protected from SCC (Fonti et al. 2025; Grüntzig et al. 2016). Previous research also highlighted that canine SCC is more likely to develop on sparsely haired, unpigmented skin or animals with white haircoats, mostly favoring the ventrum and flank (dos Santos et al. 2023; Er and Sutton 1989; Hargis et al. 1977; Madewell et al. 1981; Rothwell et al. 1987; Willcox et al. 2019). However, various alternative locations, such as hindlimb and head, have been occasionally identified (Seok and Lee 2024; Strafuss et al. 1976). Overall, no clear sex-predispositions have been recorded, although a Swiss study found that females had a lower disease risk than males, and another study identified sex-related differences in tumour locations (Er and Sutton 1989; Grüntzig et al. 2016; Kim et al. 2020; Mukaratirwa et al. 2005; Rothwell et al. 1987). Other potential contributors to canine cSCC include papillomavirus infection and previous actinic skin damage (Medeiros-Fonseca et al. 2023; Willcox et al. 2019; Zaugg et al. 2005).

Canine digital SCC, although broadly classified into the cSCC group, has different properties. It is more aggressive, has higher metastatic potential, and is more common in large and giant dog breeds with dark haircoats, such as schnauzers (Aupperle-Lellbach et al. 2023; Belluco et al. 2013; Cerezo-Echevarria et al. 2020; Madewell et al. 1982; O’Brien et al. 1992; Wobeser et al. 2007).

Despite SCC’s clinical relevance, a very limited number of large-scale Australian studies have been produced, with most being completed several decades ago. Considering that canine squamous cell carcinomas have been found to have higher occurrence rates in tropical countries, opportunities to gain access to relevant Australian data would be highly valuable for both oncological pathological analysis and clinical disease control (Alves et al. 2022). As previous research hypothesized that both intrinsic (age, breed, coat colour) and environmental (UV light exposure) factors significantly influence the development of canine cSCC, this study aimed to estimate its frequency in Australia, examine its geographic distribution and association with UV index level, describe its anatomic distribution, and identify animal-level risk factors associated with its occurrence.

## Materials and methods

### Data source

The data for this study were extracted from VetCompass Australia, which is a collection of electronic medical records from participating general practice veterinary clinics and specialist referral hospitals across the country. The VetCompass database was searched with specific search terms to find veterinary records entered between 1st January 2014 and 31st December 2023. The database contained clinical records from all major capital cities of Australia except for Darwin and Hobart. Information obtained from the database included age at diagnosis (date of diagnosis minus birth date), sex (including neuter status), coat colour, breed, clinic postcode, statistical area codes, and anatomical location of the neoplasia.

### Case selection and inclusion criteria

**Fig. 1:**
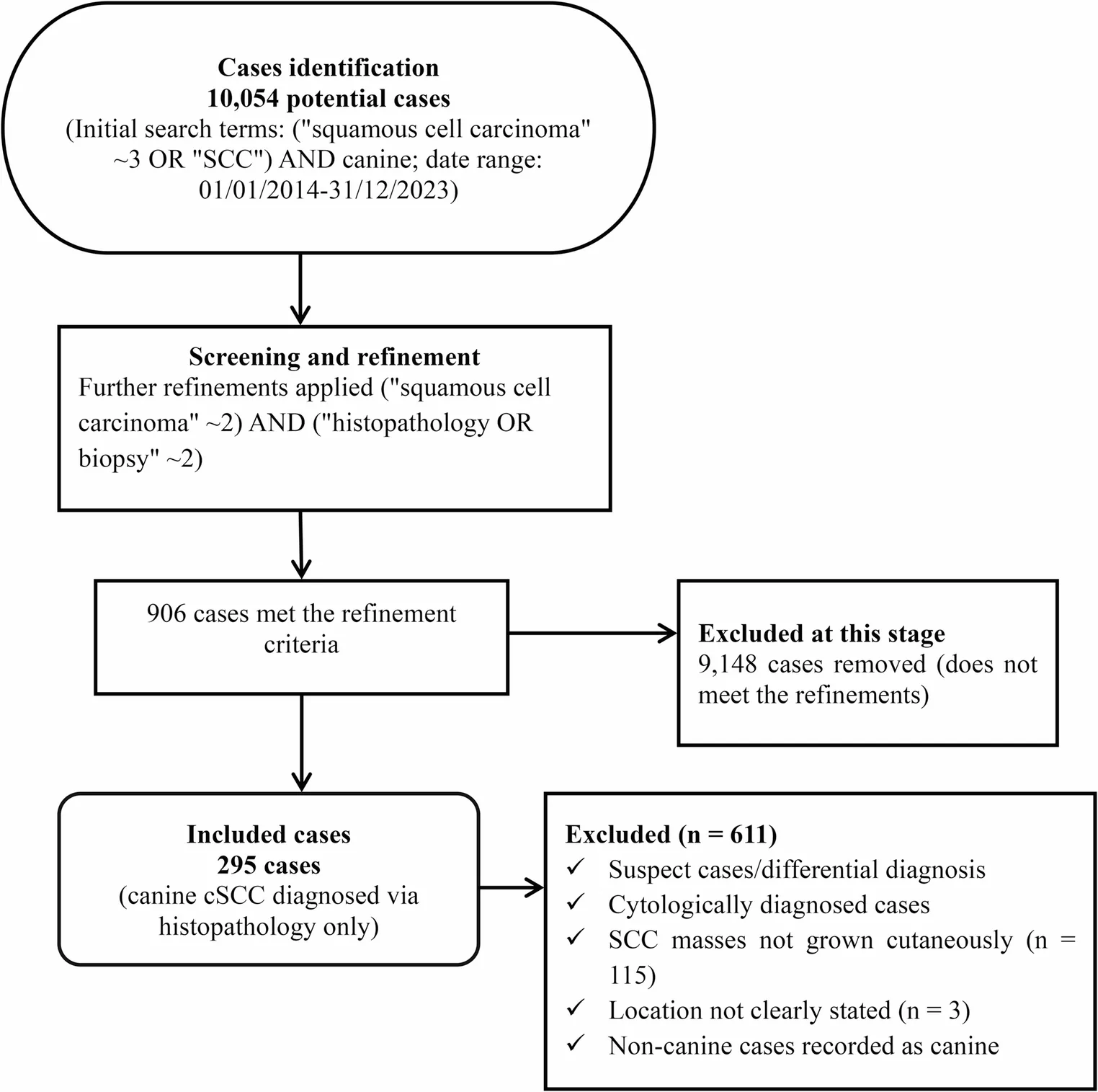
Flowchart of the selection process for the canine cutaneous squamous cell carcinoma cases

The search terms with corresponding abbreviated adjustments were used to identify canine patients diagnosed with cSCC via histopathology. The initial search produced 906 matches from the database and patients were eligible for inclusion if they were histopathologically diagnosed with cSCC between 1st January 2014 and 31st December 2023. A total of 295 canine patients met the inclusion criteria and were selected as cases (Fig. 1).

### Control selection

**Fig. 2:**
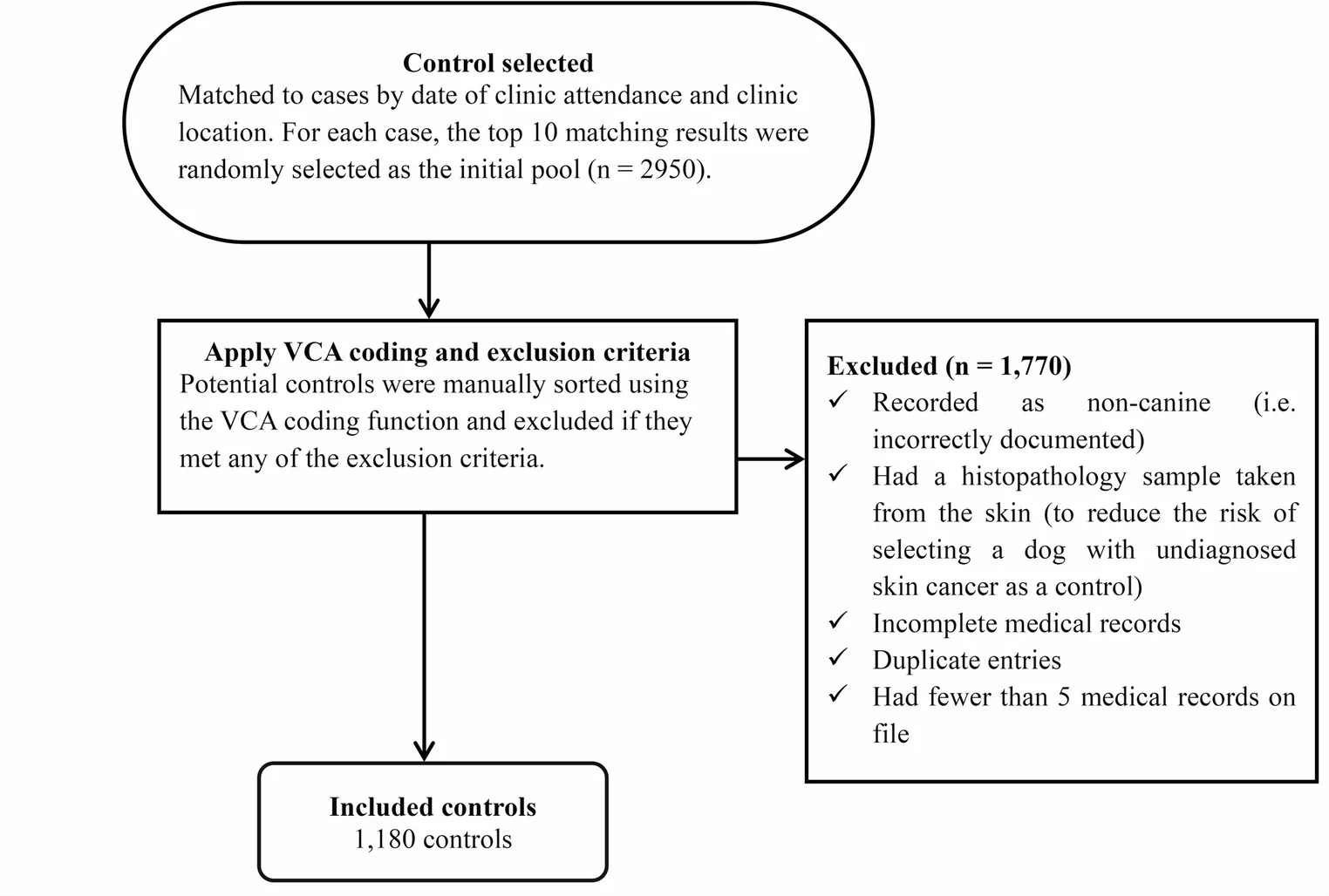
Flowchart of the selection process for the control group

Dogs were eligible as controls if they had a clinical record in the VetCompass database during the ten-year study period. For each cSCC case, up to 10 control dogs were randomly selected by matching date of clinic attendance and clinic location with control records entered within a comparable timeframe. Control dogs with fewer than five medical records were excluded, as well as duplicates and dogs that had incomplete documentation. This reduced the number of control dogs available. Thus, for each case, four matched controls were selected, resulting in a total of 1,180 controls selected for the study (Fig. 2).

### Animal level risk factors

Animal level risk factors included age, breed, sex, neuter status, coat colour, tumour anatomical location, and geographical location with corresponding UV level. If information on a risk factor was missing, it was denoted as ‘not recorded’.

#### Age

Age was grouped into 0–1 (puppy and juvenile), 2–6 (mature), 7–11 (senior), and 12+ (geriatric) years old based on the categorization method by Harvey (2021). Due to the lack of clinical cases in the 0–1 years category, it was excluded from logistic analysis to produce more representative results.

#### Breed

Breed names recorded in the VetCompass database were used in this study. Breeds with high naming similarities (e.g. miniature poodle/standard poodle, miniature schnauzer/standard schnauzer/giant schnauzer, English Staffordshire bull terrier/American Staffordshire bull terrier) were collectively grouped (e.g. both English and American Staffordshire bull terriers were combined into Staffordshire bull terriers) to facilitate larger groups for the risk factor analysis. Similarly, pure breeds with more than 10 cSCC cases were analysed individually, while those with fewer than 10 cases were grouped collectively. Mixed breeds were analysed separately as a group. The bulldog breeds (American bulldog, Australian bulldog, British bulldog, French bulldog and Johnson American bulldog) were also grouped for the risk factor analysis.

A second breed classification method, based on Dogs Australia breed groups, was used to categorize both case and control individuals for the descriptive analysis (Dogs Australia 2025). Here, dogs were classified as crossbred dogs, toy dogs, terriers, gundogs, hounds, working dogs, utility dogs, and non-sporting dogs. This additional classification enabled cross-comparison and facilitated pattern recognition.

#### Sex and neuter status

The sex and neutering status information as recorded in the VetCompass database were used in the data analysis. The relevant categorizations included male desexed, male entire, female desexed and female entire.

#### Coat colour

Coat colour classifications were performed based on the standardized nomenclature of coat colours in dogs from the Federation Cynologique Internationale (Denis 2009). The specific groups were white (dogs with solid white hair coats only), dark solid (dogs with solid black or brown hair coats only), white mixed (dogs with mixed coat colours including white), and others (all other hair coats, including other solid colours, and mixed and modified coats). Such categorizations were made in consideration of the previously identified canine SCC risks associated with coat colour and pigmentation.

#### Tumour location

The anatomical tumour locations for all cases were grouped into head, thorax, body (caudal to thorax), limb, and digit/nailbed, with tumours in any other location (ventral tail, mammary region, and submandibular area) collectively categorized as ‘other’.

#### Geographical location

The cSCC cases were summarized at two geographic levels: the Australian state/territory level and the Greater Capital City Statistical Area (GCCSA) level, based on geographical information obtained from the VetCompass Australia database. The dataset contained Statistical Area Level 2 (SA2) information corresponding to the geographic areas of the dog owners. Therefore, the geographical information used in this study represented the owners’ residential areas rather than the locations of the veterinary clinics where the dogs received veterinary care. The SA2 information enabled cases to be grouped and summarised geographically and subsequently aggregated to the state/territory and GCCSA levels. Separating the data in this way allowed for comparisons of statewide and major metropolitan area patterns.

#### UV-exposure proxies

To determine UV-exposure proxies, anatomical sites were classified as UV-exposed (head, limbs, thorax, and body caudal to the thorax) or non-UV-exposed (digit/nailbed and other locations), reflecting the recognised predilection of canine cSCC for sun-exposed, lightly pigmented, or thinly-haired skin (Vilar-Saavedra and Kitchell 2014; Veterinary Society of Surgical Oncology 2025). For coat-based UV susceptibility, white and white mixed coats were categorised as highly UV-susceptible, whereas dark solid and other coat colours were classified as less UV-susceptible, based on the protective role of pigmentation against UV radiation (Veterinary Society of Surgical Oncology 2025). This UV-proxy classification was then applied to interpret frequency patterns in the anatomical distribution of lesions and coat-colour characteristics among affected dogs.

In addition, to compare clinical data with environmental UV levels, clinic locations where cases had been seen were grouped by major Australian cities (Adelaide, Brisbane, Gold Coast, Melbourne, Perth and Sydney). Clinics outside these cities were excluded, yielding *n* = 135 for this analysis. City-level UV index data were obtained from ARPANSA and matched to corresponding case and control locations. All available noon (12:00 PM) UV index values for each city were extracted, and the mean noon UV index was used as the representative environmental exposure measure. UV index categories followed the ARPANSA scale: low (1–2), moderate (3–5), high (6–7), very high (8–10), and extreme (11+) (Australian Radiation Protection and Nuclear Safety Agency 2025).

### Statistical analysis

Descriptive analyses were performed in R (R Core Team 2025). Demographic and clinical variables for cases and controls were summarised using *tidyr* for data reshaping (Wickham et al. 2025), *tidyverse* for data manipulation and visualisation (Wickham et al. 2019), and *DescTools* for descriptive statistics, including proportions and 95% confidence intervals (Signorell 2025). Categorical variables were reported as counts and percentages with 95% confidence intervals.

The spatial distribution of cSCC frequency was mapped across Australian Statistical Area Level 2 (SA2) regions in QGIS 3.20. To further characterise spatial variation, kernel smoothing was applied in R 4.5.1 using raster() (Pebesma 2018) and ks() (Duong 2025). This produced a continuous smoothed surface of cSCC frequency, allowing examination of broader spatial trends. Bandwidth was estimated using the plug-in method (Hpi) and then doubled to provide additional smoothing in the final kernel density estimation. The final raster output illustrated estimated spatial intensity per SA2 region.

Risk factor analyses were conducted on the individual-dog level using a matched case-control design, with each cSCC case paired with four control dogs (Henning et al. 2009). Conditional logistic regression was performed using *survival*::clogit() (Therneau 2024) to assess risk factors associated with cSCC, applying conditional likelihood appropriate for matched data (Gail et al. 1981). Potential risk factors included sex, sex/neuter status, breed category, coat-colour group and age group, and were processed using *broom*::tidy() (Robinson et al. 2025). Model outputs were presented as odds ratios (OR) with 95% confidence intervals (CI) and p-values using *gtsummary*::tbl_regression(exponentiate = TRUE) (Sjoberg et al. 2021). Variables with *p* ≤ 0.25 in univariable analyses (Henning et al. 2009) were selected in multivariable modelling. Model diagnostics included likelihood ratio tests for nested models and AIC/BIC for non-nested model comparison. Multicollinearity was assessed using variance inflation factors (VIFs) from the *car* package (Fox et al. 2024), with VIF < 5 used as the threshold for acceptable collinearity (O’Brien 2007). Statistical significance was defined as *p* ≤ 0.05. A sensitivity analysis was performed by excluding digital/nailbed SCC cases (*n* = 51) and their matched controls and repeating all conditional logistic regression analyses.

## Results

### Characteristics of the canine cutaneous squamous cell carcinoma cases

Among the cSCC cases, the majority were senior dogs (58.6%; 95% CI: 53.0-64.1). A substantial proportion of cases also involved desexed male dogs (45.1%; 95% CI: 39.5–50.8), breeds represented by fewer than 10 cases (34.2%; 95% CI: 29.1–39.8), and dogs with white mixed coat colours (43.7%; 95% CI: 38.2–49.4) (Table 1).

**Table 1:** Demographic characteristics of 295 cutaneous squamous cell carcinoma cases in Australian dogs

Variable	Category	Frequency	Percentage (95% CI)
Age	Mature (2–6 yrs)	69	23.4 (18.9–28.5)
	Senior (7–11 yrs)	173	58.6 (53.0-64.1)
	Geriatric (12 + yrs)	53	18.0 (14.0-22.8)
Sex	Female	143	48.5 (42.8–54.2)
	Male	152	51.5 (45.8–57.2)
Neuter status	Female intact	14	4.8 (2.9–7.8)
	Female desexed	129	43.7 (38.2–49.4)
	Male intact	19	6.4 (4.2–9.8)
	Male desexed	133	45.1 (39.5–50.8)
Breed	Mixed breed	83	28.1 (23.3–33.5)
	Bulldog	23	7.8 (5.3–11.4)
	Dalmatian	20	6.8 (4.4–10.2)
	Jack Russell terrier	10	3.4 (1.9–6.1)
	Other pure breeds with < 10 cases	101	34.2 (29.1–39.8)
	Schnauzer	10	3.4 (1.9–6.1)
	Staffordshire bull terrier	48	16.3 (12.5–20.9)
Breed group	Crossbred dogs	94	31.8 (26.8–37.4)
	Toy dogs	4	1.4 (0.5–3.4)
	Terriers	73	24.8 (20.2–29.9)
	Gundogs	21	7.1 (4.7–10.6)
	Hounds	18	6.1 (3.9–9.4)
	Working dogs	12	4.1 (2.3–6.9)
	Utility dogs	28	9.5 (6.7–13.4)
	Non-sporting dogs	45	15.2 (11.6–19.8)
Coat colour	Dark solid	29	9.8 (6.9–13.8)
	White	50	17.0 (13.1–21.7)
	White mixed	129	43.7 (38.2–49.4)
	Other	87	29.5 (24.6–34.9)

CI: Confidence interval

A total of 48 canine breeds, including mixed breeds as a collective category, were diagnosed with cSCC in this study. The highest proportion of cases occurred in mixed-breed dogs (*n* = 83; 28.1%), followed by Staffordshire bull terriers (*n* = 48; 16.3%), Dalmatians (*n* = 20; 6.8%), and American bulldogs (*n* = 15; 5.1%) (Online Resource 1). The most common location for cSCC was in body parts caudal to the thorax (54.9%; 95% CI: 49.2–60.5) (Table 2). In contrast, the lowest frequency was recorded in the category labelled other, which included the ventral tail, mammary region, and submandibular area (1.7%; 95% CI: 0.7–3.9) (Table 2).

**Table 2:** Frequency distributions of cutaneous squamous cell carcinoma across anatomical locations in 295 Australian dogs

Anatomical location	Frequency	Percentage (95% CI)
Body caudal to thorax	162	54.9 (49.2–60.5)
Digit/nailbed	51	17.3 (13.4–22.0)
Head	27	9.2 (6.4–12.9)
Limbs	19	6.4 (4.2–9.8)
Not recorded	17	5.8 (3.6-9.0)
Thorax	14	4.7 (2.9–7.8)
Other	5	1.7 (0.7–3.9)

CI: Confidence interval

### Spatial distribution of canine cutaneous squamous cell carcinoma in Australia

Table 3 shows the distribution of cSCC cases across the Australian states/territories, and greater capital city statistical areas. The highest proportions of cases were observed in Queensland (39.7%; 95% CI: 34.0-45.5) and New South Wales (39.3%; 95% CI: 33.7–45.2). At the level of greater capital territory regions, the highest percentage of cSCC cases occurred in the rest of Queensland (24.1%; 95% CI: 19.3–29.4), followed by the rest of New South Wales (22.7%; 95% CI: 18.1–27.9), greater Sydney (16.6%; 95% CI: 12.6–21.4), and greater Brisbane (15.6%; 95% CI: 11.7–20.3).

**Table 3:** Distribution of 295 canine cutaneous squamous cell carcinoma cases by Australian state/territory, and by greater capital city statistical area

Variable	Category	Frequency	Percentage (95% CI)
State/territory	Queensland	117	39.7 (34.0-45.5)
	New South Wales	116	39.3 (33.7–45.2)
	Victoria	37	12.5 (9.0-16.9)
	Australian Capital Territory	10	3.4 (1.6–6.2)
	Western Australia	8	2.7 (1.2–5.3)
	South Australia	7	2.4 (0.9–4.8)
Greater capital city statistical area	Rest of Queensland	71	24.1 (19.3–29.4)
	Rest of New South Wales	67	22.7 (18.1–27.9)
	Greater Sydney	49	16.6 (12.6–21.4)
	Greater Brisbane	46	15.6 (11.7–20.3)
	Greater Melbourne	31	10.5 (7.3–14.6)
	Australian Capital Territory	10	3.4 (1.6–6.2)
	Greater Perth	8	2.7 (1.2–5.3)
	Rest of Victoria	6	2.0 (0.8–4.4)
	Greater Adelaide	5	1.7 (0.6–3.9)
	Rest of South Australia	2	0.7 (0.1–2.4)

CI: Confidence interval

**Fig. 3:**
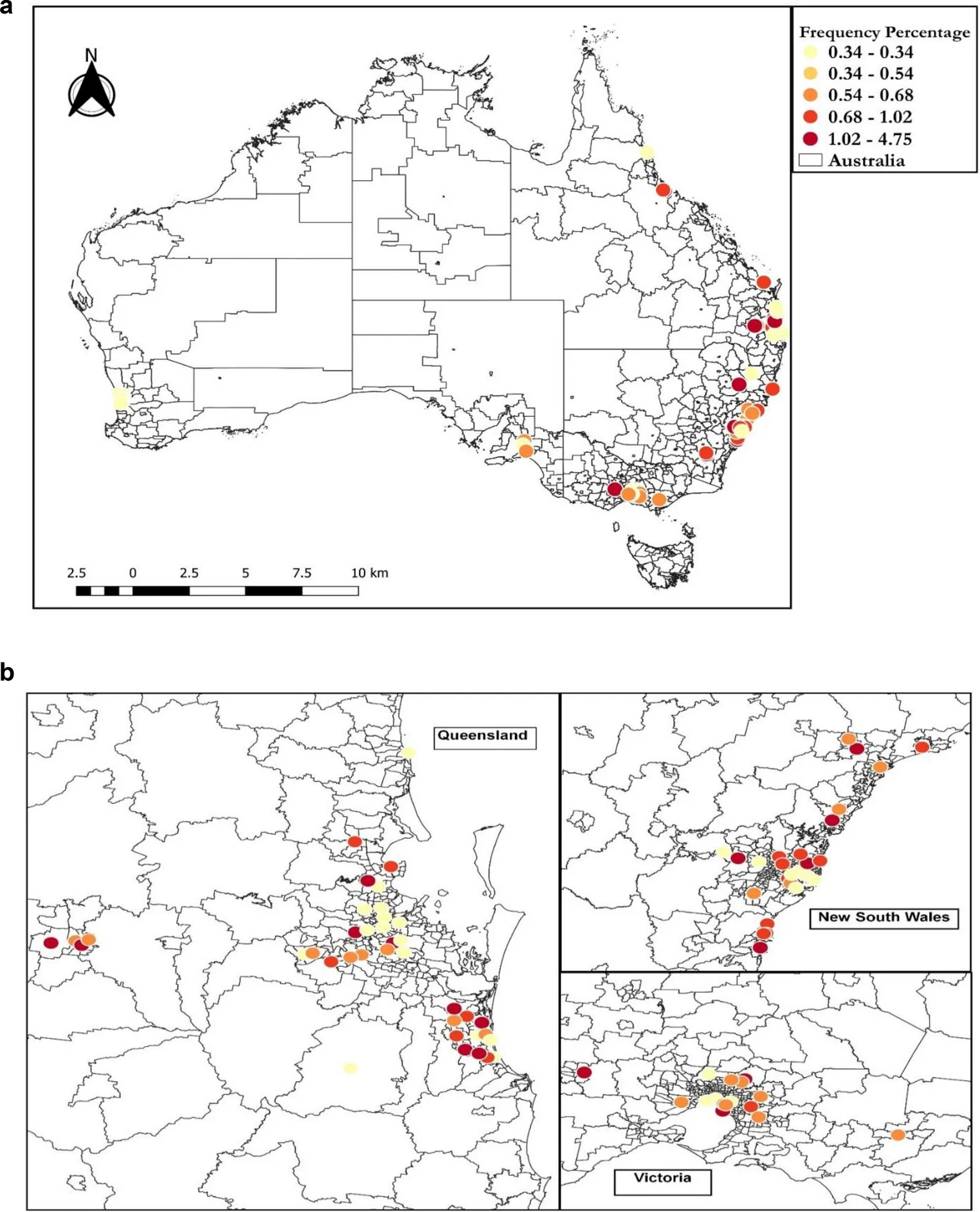
Spatial distribution of canine cutaneous squamous cell carcinoma frequency across SA2 regions in (**a**) Australia and (**b**) Queensland, New South Wales and Victoria

In this study, 110 SA2 regions across Australia reported cases of cSCC. Most cases occurred in Queensland, followed by New South Wales and Victoria. In addition, the spatial analysis shows that case concentrations were strongly clustered along the coastal areas of these states, indicating a geographically aggregated distribution pattern (Fig. 3).

Similarly, the kernel-smoothed frequency map in Online Resource 2 highlights distinct hotspot regions along the coastal areas of Queensland and New South Wales, indicating higher concentrations of cSCC cases in these zones.

**Fig. 4:**
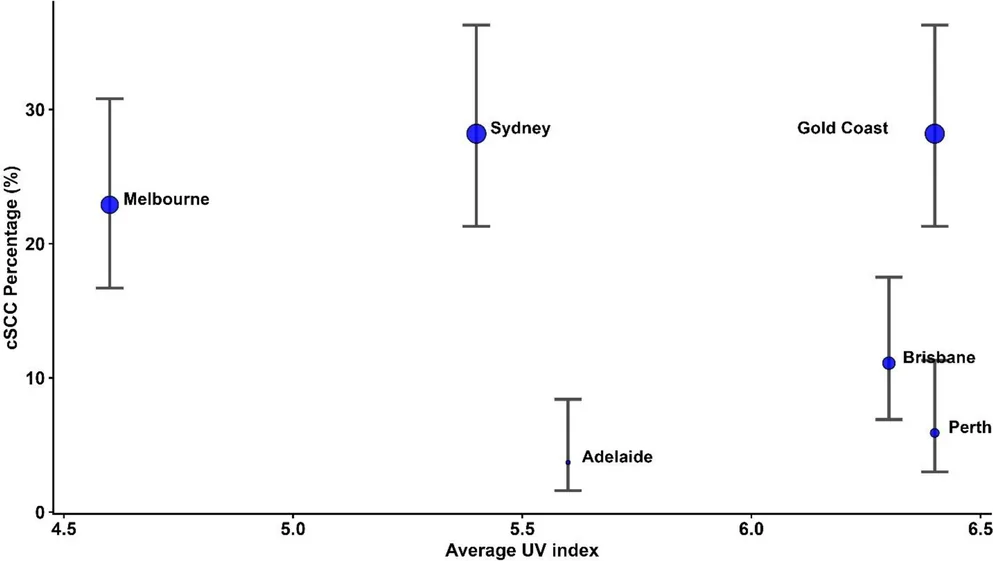
Scatter plot showing the descriptive comparison between the percentage of reported canine cutaneous squamous cell carcinoma (cSCC) cases and average annual UV index among the six Australian cities with the highest numbers of reported cases (*n* = 135 cases)

Table 4; Fig. 4 presents the reported cSCC case frequencies and average annual UV index for the six Australian cities with the highest numbers of reported cases. Gold Coast and Sydney recorded the highest numbers of cSCC cases. The average annual UV index varied across the selected cities, with the highest values observed in the Gold Coast and Perth and the lowest in Melbourne. The scatter plot was used to visually compare case frequencies with average UV index levels; no statistical test of association was performed. Therefore, these findings should be interpreted as descriptive observations rather than evidence of an association between UV exposure and cSCC occurrence.

**Table 4:** Canine cutaneous squamous cell carcinoma case frequency (*n* = 135) and UV index across major Australian cities

City	Frequency	Percentage (95% CI)	Average UV index
Gold Coast	38	28.2 (21.3–36.3)	6.4
Sydney	38	28.2 (21.3–36.3)	5.4
Melbourne	31	22.9 (16.7–30.8)	4.6
Brisbane	15	11.1 (6.9–17.5)	6.3
Perth	8	5.9 (3.0-11.3)	6.4
Adelaide	5	3.7 (1.6–8.4)	5.6

CI: Confidence interval

When cities were classified using ARPANSA UV-exposure categories, all fell within the moderate (UV 3–5) or high (UV 6–7) ranges, with none in the extreme categories. A slightly greater proportion of observations occurred under moderate UV levels (*n* = 74; 54.8%) compared with high UV levels (*n* = 61; 45.2%).

### Risk factor analysis

The univariable conditional regression analysis indicated that age was a strong predictor of disease risk. Senior dogs had 5.7 times higher odds of developing cSCC (95% CI: 4.2–7.9), and geriatric dogs had 3.8 times higher odds (95% CI: 2.5–5.7), compared with mature dogs (*p* ≤ 0.001). Dogs with white coats exhibited 3.1 times higher odds (95% CI: 1.8–5.2), and those with white mixed coat colours showed 2.4 times higher odds (95% CI: 1.5–3.7), relative to dogs with dark solid coat colours (*p* ≤ 0.001). Additionally, Dalmatians (OR = 34.9; 95% CI: 10.2-120.3) and bulldogs (OR = 14.7; 95% CI: 6.7–32.2) had markedly higher odds of developing cSCC compared with mixed-breed dogs, and these associations were statistically significant (*p* ≤ 0.001) (Table 5). To evaluate the potential influence of digital SCC on the observed associations, a sensitivity analysis was conducted after excluding all digital/nailbed SCC cases (*n* = 51) and their matched controls. Results of the univariable and multivariable conditional logistic regression analyses are presented in Supplementary Tables S1 and S2, respectively. The findings were largely unchanged from the primary analysis.

**Table 5:** Results of the univariable conditional regression analysis examining the associations between cutaneous squamous cell carcinoma and animal-level risk factors in Australian dogs

Variable	Category	Case Freq. (%) (*n* = 295)	Control Freq. (%) (*n* = 1180)	OR (95% CI)	Wald Stat.	*p*-value
Age	Mature (2–6 yrs)	69 (9.0)	694 (91.0)	**1**	**--**	**--**
	Senior (7–11 yrs)	173 (35.0)	321 (65.0)	5.7 (4.2–7.9)	10.5	< 0.001
	Geriatric (12 + yrs)	53 (24.3)	165 (75.7)	3.8 (2.5–5.7)	6.1	< 0.001
Sex	Female	143 (19.2)	601 (80.8)	**1**	**--**	**--**
	Male	152 (20.8)	579 (79.2)	1.1 (0.9–1.4)	0.8	0.451
Neuter status	Female intact	14 (14.1)	85 (85.9)	**1**	**--**	**--**
	Female desexed	129 (20.0)	517 (80.0)	1.5 (0.8–2.8)	1.4	0.171
	Male intact	19 (16.4)	97 (83.6)	1.2 (0.6–2.5)	0.5	0.643
	Male desexed	133 (21.7)	481 (78.3)	1.7 (0.9–3.1)	1.7	0.087
Breed	Mixed breed	83 (13.5)	531 (86.5)	**1**	**--**	**--**
	Bulldog	23 (69.7)	10 (30.3)	14.7 (6.7–32.2)	6.7	< 0.001
	Dalmatian	20 (87.0)	3 (13.0)	34.9 (10.2-120.3)	5.6	< 0.001
	Jack Russell terrier	10 (23.8)	32 (76.2)	2.0 (0.9–4.3)	1.8	0.074
	Other pure breeds with < 10 cases	101 (16.1)	527 (83.9)	1.2 (0.9–1.7)	1.2	0.246
	Schnauzer	10 (34.5)	19 (65.5)	4.7 (2.0-11.1)	3.5	0.003
	Staffordshire bull terrier	48 (45.3)	58 (54.7)	5.8 (3.6–9.5)	7.2	< 0.001
Coat colour	Dark solid	29 (14.5)	171 (85.5)	**1**	**--**	**--**
	White	50 (34.5)	95 (65.5)	3.1 (1.8–5.2)	4.2	< 0.001
	White mixed	129 (28.9)	318 (71.1)	2.4 (1.5–3.7)	3.8	< 0.001
	Other	87 (13.0)	582 (87.0)	0.9 (0.6–1.4)	-0.6	0.577
	Not recorded	0 (0.0)	14 (100.0)	0.0 (0 - + infinity)	-0.03	0.996

OR: Odds ratio, OR with value of 1 represents the reference category; CI: Confidence interval

In the multivariable final model, age remained a strong predictor of cSCC occurrence. Senior dogs had significantly higher odds of developing cSCC (OR = 7.4; 95% CI: 5.0-10.8), as did geriatric dogs (OR = 4.8; 95% CI: 2.9–7.9), compared with mature dogs (*p* ≤ 0.001). Breed was also significantly associated with cSCC risk. Dalmatians (OR = 25.7; 95% CI: 6.9–95.6), bulldogs (OR = 21.2; 95% CI: 8.4–53.7), Staffordshire terriers (OR = 8.5; 95% CI: 4.8–15.2), and schnauzers (OR = 7.1; 95% CI: 2.6–19.1) exhibited markedly higher odds of cSCC occurrence compared with mixed breed dogs, with all associations statistically significant (*p* ≤ 0.001) (Table 6).

**Table 6:** Results of the final multivariable conditional regression model identifying risk factors associated with cutaneous squamous cell carcinoma occurrence in Australian dogs

Variable	Category	OR	95% CI	Wald Stat.	*p*-value
Age	Mature (2–6 yrs)	**1**	**--**	**--**	**--**
	Senior (7–11 yrs)	7.4	5.0-10.8	10. 2	< 0.001
	Geriatric (12 + yrs)	4.8	3.0-7.9	6.3	< 0.001
Breed	Mixed breed	**1**	**--**	**--**	**--**
	Bulldog	21.2	8.4–53.7	6.4	< 0.001
	Dalmatian	25.7	6.9–95.6	4.8	< 0.001
	Jack Russell terrier	1.2	0.5–2.9	0.5	0.636
	Other pure breeds with < 10 cases	1.5	1.0-2.2	2.1	0.033
	Schnauzer	7.1	2.6–19.1	3.8	< 0.001
	Staffordshire bull terrier	8.5	4.8–15.2	7.2	< 0.001
Coat colour	Dark solid	**1**	**--**	**--**	**--**
	White	2.8	1.5–5.2	3.2	< 0.001
	White mixed	2.5	1.5–4.2	3.3	< 0.001
	Other	1.1	0.6–1.9	0.3	0.765
	Not recorded	0.0	0 - + infinity	-0.01	0.996

OR: Odds ratio; OR with value of 1 represents the reference category; CI: Confidence interval

## Discussion

Cutaneous squamous cell carcinoma is a significant canine skin tumour, accounting for approximately 5% of skin tumours worldwide, with higher prevalence reported in tropical regions (Alves et al. 2022; Kukolj et al. 2021; Santos et al. 2020). UV radiation is a well-established carcinogenic factor in its pathogenesis, contributing to increased occurrence in areas with high ambient UV exposure. Given Australia’s tropical and subtropical environments and consistently high UV indices, ongoing monitoring of cSCC and evaluation of associated risk factors are essential. The findings of this study provide updated epidemiological insight to support more effective identification, intervention, and prevention.

In the current study, the anatomical distribution of cSCC showed that most cases occurred in regions caudal to the thorax (54.9%; 95% CI: 49.2–60.5), followed by the digit/nailbed (17.3%), head (9.2%), and limbs (6.4%). This distribution aligns partially with the study by Alves et al. (2022), who also reported a higher occurrence of lesions in ventral body regions. However, the current results contrast with previous studies in which most SCC cases were reported in the forelimbs (Belluco et al. 2013; Marconato et al. 2021) and oral cavity (Strafuss et al. 1976). In humans, most cSCC cases develop on the head and neck (Bibee et al. 2020) and pinna of the ear (Nuño-González et al. 2012). The discrepancies between studies are likely due to differences in study design, sampling strategies, and the anatomical sites included, as some investigations focused exclusively on specific body regions.

The frequency of cSCC across animal-level risk factors showed that most cases occurred in senior dogs aged seven to 11 years old, (58.6%), males (51.5%), desexed males (45.1%), mixed breeds (28.1%), and white mixed-coloured dogs (43.7%). The current findings contrast with studies that reported a higher frequency of cSCC in dark-coated dogs (Belluco et al. 2013; Marconato et al. 2021). However, studies in humans have shown that cSCC is more common in fair-skinned individuals (Parekh and Seykora 2017; Waldman and Schmults 2019), which aligns with the association between lighter pigmentation and increased UV sensitivity. The current results are consistent with previous reports indicating that cSCC is more frequent in castrated male dogs (Raleigh et al. 2025) and older dogs (Belluco et al. 2013; Strafuss et al. 1976). Similarly, previous studies have reported a relatively high frequency of cSCC cases among mixed-breed dogs (Strafuss et al. 1976). In addition, human studies have shown a higher burden of cSCC among men and older adults (Green and Olsen 2017; Olsen et al. 2024; Parekh and Seykora 2017; Waldman and Schmults 2019). In contrast, other reports have identified breed-specific predispositions, including schnauzers (Marconato et al. 2021), German shepherds (Strafuss et al. 1976), and golden retrievers (Raleigh et al. 2025). The discrepancies between studies might be due to differences in study population characteristics, sample size, and the breeds represented in each dataset. Additionally, the higher frequency of cSCC in older dogs is biologically plausible, as age-related declines in immune function and cumulative UV exposure increase susceptibility to carcinogenesis.

The spatial distribution analysis showed that New South Wales and Queensland were associated with higher cSCC case frequencies than Victoria, the Australian Capital Territory, Western Australia and South Australia. In addition, most cases were reported from coastal areas within these states. The kernel-smoothed spatial distribution further supported this pattern. The higher frequency observed in New South Wales and Queensland may be partly explained by higher pet population densities and absolute dog numbers, as well as better access to veterinary services. These states also experience high ambient UV radiation levels for much of the year, particularly during summer months. While UV exposure was assessed using regional average UV data rather than individual-level measurements, the observed geographic patterns are consistent with a potential contribution of UV radiation to cSCC risk through cumulative UV-induced skin damage. However, the observed spatial pattern may also reflect regional differences in VetCompass coverage and veterinary service utilisation. Ultraviolet light is a very well-established carcinogen in dogs and many other mammalian species (Willcox et al. 2019). Associations between UV exposure and increased cSCC risk have also been consistently reported in human studies (Green and Olsen 2017; Mueller et al. 2019; Olsen et al. 2024; Waldman and Schmults 2019). Therefore, our findings are consistent with a possible role for UV exposure in canine cSCC development, although further studies incorporating individual-level exposure data are needed to better define this relationship.

The multivariable conditional regression analysis showed that the odds of cSCC occurrence were significantly higher in senior dogs aged seven to 11 years (OR = 7.4; 95% CI: 5.0-10.8) and in geriatric dogs aged 12 years and older (OR = 4.8, 95% CI: 3.0-7.9) compared with mature dogs aged two to six years (*p* ≤ 0.001). These findings align with previous studies reporting a higher frequency of cSCC in older dogs (Belluco et al. 2013; Strafuss et al. 1976) and in older adults in human populations (Green and Olsen 2017; Olsen et al. 2024; Parekh and Seykora 2017; Waldman and Schmults 2019). This association between advancing age and increased oncogenic risk is consistent with the cumulative DNA and cellular damage caused by prolonged UV light exposure (Villamil et al. 2011). The absence of cases in dogs younger than two years further supports the understanding of cSCC as a disease driven by chronic environmental ultraviolet exposure and long latency, rather than an acute or developmental process.

In the current study, the odds of cSCC occurrence were significantly higher in Dalmatians, bulldogs, Staffordshire bull terriers, and schnauzers compared with mixed-breed dogs. These findings support previous reports, although substantial variation in breed susceptibility has been documented across studies (Er and Sutton 1989; Fonti et al. 2025; Grüntzig et al. 2016; Marconato et al. 2021; Rothwell et al. 1987; Tompkins et al. 2020). The higher odds of occurrence observed in Dalmatians, Staffordshire bull terriers, and bulldogs seen in this study may be attributable to phenotypic traits such as short haircoats, sparsely haired ventral skin, and, particularly in Dalmatians, lightly pigmented coats. These features are likely to reduce photoprotection and increase susceptibility to UV-induced carcinogenesis. In schnauzers, increased KITLG gene expression has been previously linked to digital SCC development (García-Reynoso et al. 2025), which may contribute to the elevated risk observed here. Additionally, lifestyle and environmental factors, including outdoor activity patterns and cumulative UV exposure, may further influence breed-specific risk profiles.

The multivariable conditional logistic regression analysis also showed that the odds of cSCC occurrence were significantly higher in white coloured dogs (OR = 2.8; 95% CI: 1.5–5.2) and white mixed-coloured dogs (OR = 2.5; 95% CI: 1.5–4.2) compared with dark solid-coloured dogs (*p* ≤ 0.001). This finding aligns with earlier studies demonstrating that lightly pigmented haircoats are a well-established risk factor for canine SCC development (Er and Sutton 1989; Rothwell et al. 1987; Willcox et al. 2019). Lightly pigmented coats contain lower melanin, which is the primary photoprotective pigment that absorbs UV radiation and mitigates oxidative DNA damage (Slominski et al. 2022). Studies in humans have shown that cSCC is more common in fair-skinned individuals (Parekh and Seykora 2017; Waldman and Schmults 2019), further supporting the link between reduced pigmentation and increased vulnerability to UV-induced carcinogenesis. However, the overall study population was relatively small, resulting in limited subgroup sizes, wide confidence intervals, unstable odds ratio estimates and reduced precision. These factors should be considered key limitations of the current study. Additionally, cases and controls were not matched by demographic factors, which may have introduced confounding. Despite these constraints, the findings still provide meaningful insight into the factors influencing cSCC development in dogs in the Australian context. The consistency of findings following exclusion of digital SCC cases suggests that inclusion of digital and non-digital SCCs together did not materially influence the principal conclusions of the study. A limitation of this study is that individual-level UV exposure data were unavailable, limiting the assessment of UV exposure in the multivariable analysis.

In conclusion, the body caudal to thorax emerged as the most affected anatomical region, aligning with patterns observed in sun-exposed skin. Spatial analysis further revealed that cases were more frequently reported in New South Wales and Queensland, particularly within coastal regions. These findings provide descriptive information on the anatomical and geographical distribution of cSCC in dogs. This study also demonstrated that breed, age, and coat pigmentation are significant risk factors for cSCC in dogs. Senior and geriatric dogs, dogs with lightly pigmented coats, and several specific breeds showed markedly increased odds of developing cSCC. Further research incorporating individual patient tracking and direct measurement of UV exposure levels is recommended to better characterise environmental factors that may contribute to cSCC development in dogs.

## Supplementary Information

Below is the link to the electronic supplementary material.

Supplementary Material 1 (PDF 203 KB)

Supplementary Material 2 (PDF 198 KB)

## Data Availability

Restrictions apply to the availability of the data analysed in this study, which were used with permission from VetCompass Australia, and so the data are not publicly available.
