# Supplementary material for: Demographic and environmental factors associated with canine cutaneous squamous cell carcinoma development in Australia: a retrospective matched case-control analysis
Source: Vet Res Commun. 2026 Oct 24;51(1):20. doi: 10.1007/s11259-026-11595-5 (PMC13695678; doi:10.1007/s11259-026-11595-5)
Supplement: Supplementary file 1 [file 11259_2026_11595_MOESM1_ESM.pdf]

Supplementary Table S1: Sensitivity analysis: Univariable conditional logistic regression analyses of animal-level risk factors associated with non-digital cutaneous squamous cell carcinoma in Australian dogs.

Variable	Category	Case Freq. (%) (n = 244)	Control Freq. (%) (n = 976)	OR (95% CI)	Wald Stat.	p-value
Age	Mature (2-6 yrs)	66 (10.2)	584 (89.8)	**1**	--	--
	Senior (7-11 yrs)	141 (35.2)	260 (64.8)	5.0 (3.5-7.0)	9.2	< 0.001
	Geriatric (12+ yrs)	37 (21.9)	132 (78.1)	2.8 (1.8-4.5)	4.3	< 0.001
Sex	Female	123 (19.5)	507 (80.5)	**1**	--	--
	Male	121 (20.5)	469 (79.5)	1.1 (0.8-1.4)	0.4	0.668
Neuter status	Female intact	12 (13.5)	77 (86.5)	**1**	--	--
	Female desexed	111 (20.5)	431 (79.5)	1.6 (0.9-3.1)	1.5	0.129
	Male intact	15 (15.6)	81 (84.4)	1.2 (0.5-2.5)	0.5	0.643
	Male desexed	106 (21.5)	387 (78.5)	1.8 (0.9-3.3)	1.7	0.088
Breed	Mixed breed	69 (13.6)	438 (86.4)	**1**	--	--
	Bulldog	23 (71.9)	9 (28.1)	14.6 (6.4-33.0)	6.4	< 0.001
	Dalmatian	20 (87.0)	3 (13.0)	30.9 (9.0-106.1)	5.5	< 0.001
	Jack Russell terrier	10 (27.5)	26 (72.2)	2.5 (1.1-5.5)	2.2	0.027
	Other pure breeds with < 10 cases	73 (14.5)	432 (85.5)	1.1 (0.7-1.5)	0.3	0.767
	Schnauzer	2 (11.1)	16 (88.9)	1.4 (0.3-6.4)	0.4	0.673
	Staffordshire bull terrier	47 (47.5)	52 (52.5)	5.9 (3.6-9.8)	6.9	< 0.001
Coat colour	Dark solid	7 (4.8)	138 (95.2)	**1**	--	--
	White	49 (40.2)	73 (59.8)	14.1 (6.0-33.1)	6.0	< 0.001
	White mixed	127 (32.4)	265 (67.6)	10.0 (4.5-22.3)	5.6	< 0.001
	Other	61 (11.1)	488 (88.9)	2.6 (1.1-5.8)	2.3	0.023
	Not recorded	0 (0.0)	12 (100.0)	0.0 (0.0 - + infinity)	0.0	0.996

OR: Odds ratio; OR with value of 1 represents the reference category; CI: Confidence interval
