# Supplementary material for: Demographic and environmental factors associated with canine cutaneous squamous cell carcinoma development in Australia: a retrospective matched case-control analysis
Source: Vet Res Commun. 2026 Oct 24;51(1):20. doi: 10.1007/s11259-026-11595-5 (PMC13695678; doi:10.1007/s11259-026-11595-5)
Supplement: Supplementary file 2 [file 11259_2026_11595_MOESM2_ESM.pdf]

Supplementary Table S2: Sensitivity analysis: Final multivariable conditional logistic regression model of risk factors associated with non-digital cutaneous squamous cell carcinoma in Australian dogs.

Variable	Category	OR	95% CI	Wald Stat.	p-value
Age	Mature (2-6 yrs)	**1**	--	--	--
	Senior (7-11 yrs)	6.1	4.0-9.3	8.4	< 0.001
	Geriatric (12+ yrs)	3.4	2.0-6.0	4.3	< 0.001
Breed	Mixed breed	**1**	--	--	--
	Bulldog	16.1	6.1-42.2	5.7	< 0.001
	Dalmatian	19.9	5.3-74.8	4.4	< 0.001
	Jack Russell terrier	1.5	0.6-3.6	0.9	0.378
	Other pure breeds with < 10 cases	1.3	0.8-2.0	1.1	0.253
	Schnauzer	2.6	0.5-14.7	1.1	0.286
	Staffordshire bull terrier	9.0	4.9-16.8	7.0	< 0.001
Coat colour	Dark solid	**1**	--	--	--
	White	11.7	4.5-30.7	5.0	< 0.001
	White mixed	10.0	4.1-24.3	5.0	< 0.001
	Other	3.2	1.3-7.8	2.5	0.013
	Not recorded	0.0	0.0 - + infinity	0.0	0.996

OR: Odds ratio; OR with value of 1 represents the reference category; CI: Confidence interval
